# Lipocalin-2 in Fructose-Induced Fatty Liver Disease

**DOI:** 10.3389/fphys.2017.00964

**Published:** 2017-11-28

**Authors:** Jessica Lambertz, Thorsten Berger, Tak W. Mak, Josef van Helden, Ralf Weiskirchen

**Affiliations:** ^1^Institute of Molecular Pathobiochemistry, Experimental Gene Therapy and Clinical Chemistry, RWTH University Hospital Aachen, Aachen, Germany; ^2^The Campbell Family Institute for Breast Cancer Research, University Health Network, Toronto, ON, Canada; ^3^Ontario Cancer Institute, University Health Network, Toronto, ON, Canada; ^4^MVZ Dr. Stein und Kollegen, Moenchengladbach, Germany

**Keywords:** fructose, diet, liver, steatosis, fat, lipocalin 2, NAFLD

## Abstract

The intake of excess dietary fructose most often leads to non-alcoholic fatty liver disease (NAFLD). Fructose is metabolized mainly in the liver and its chronic consumption results in lipogenic gene expression in this organ. However, precisely how fructose is involved in NAFLD progression is still not fully understood, limiting therapy. Lipocalin-2 (LCN2) is a small secreted transport protein that binds to fatty acids, phospholipids, steroids, retinol, and pheromones. LCN2 regulates lipid and energy metabolism in obesity and is upregulated in response to insulin. We previously discovered that LCN2 has a hepatoprotective effect during hepatic insult, and that its upregulation is a marker of liver damage and inflammation. To investigate if LCN2 has impact on the metabolism of fructose and thereby arising liver damage, we fed wild type and *Lcn2*^−/−^ mice for 4 or 8 weeks on diets that were enriched in fructose either by adding this sugar to the drinking water (30% w/v), or by feeding a chow containing 60% (w/w) fructose. Body weight and daily intake of food and water of these mice was then measured. Fat content in liver sections was visualized using Oil Red O stain, and expression levels of genes involved in fat and sugar metabolism were measured by qRT-PCR and Western blot analysis. We found that fructose-induced steatosis and liver damage was more prominent in female than in male mice, but that the most severe hepatic damage occurred in female mice lacking LCN2. Unexpectedly, consumption of elevated fructose did not induce *de novo* lipogenesis or fat accumulation. We conclude that LCN2 acts in a lipid-independent manner to protect the liver against fructose-induced damage.

## Introduction

Non-alcoholic fatty liver disease (NAFLD) comprises a disease spectrum that includes steatosis (fatty liver), non-alcoholic steatohepatitis (NASH), and cirrhosis. While simple steatosis is reversible, NASH is characterized by hepatocyte injury, inflammation and fibrosis that can escalate to cirrhosis, liver failure, and even hepatocellular carcinoma (de Alwis and Day, 2008). NAFLD is associated with features of metabolic syndrome, which include abdominal obesity, insulin resistance, glucose intolerance or type 2 diabetes mellitus, and dyslipidemia. Therefore, NAFLD is often described as the hepatic manifestation of metabolic syndrome (Marchesini et al., 2003; Kotronen and Yki-Järvinen, 2008; Ratziu et al., 2010; Vanni et al., 2010). Diets enriched in saturated fat, soft drinks or fatty meats, or diets deficient in fiber, anti-oxidants or omega 3 fatty acids, have all been connected to an increased risk of NAFLD (Zelber-Sagi et al., 2007; Yasutake et al., 2014).

Lipocalin 2 (LCN2), or neutrophil gelatinase-associated lipocalin (NGAL), is a small secreted protein belonging to the lipocalin family. LCN2 binds to small hydrophobic molecules, including fatty acids, phospholipids, steroids, retinol and pheromones, and thereby controls their transport, stability and release (Flower et al., 2000; Schlehuber and Skerra, 2005; Zhou and Rui, 2010; Sultan et al., 2013). Accordingly, LCN2 plays roles in organogenesis, cell differentiation, cell migration, apoptosis, and inflammation (Devireddy et al., 2005; Bachman et al., 2009; Borkham-Kamphorst et al., 2011, 2013; Chakraborty et al., 2012; Wilson et al., 2016). In one experimental NASH model in mice, LCN2 upregulation was found in hepatic parenchymal cells positioned next to inflammatory cell clusters, making LCN2 a putative NASH marker. Elevated LCN2 has also been correlated with hepatic inflammation in NAFLD (Semba et al., 2013). Previously, we discovered that high levels of LCN2 are associated with acute and inflammatory liver damage (Borkham-Kamphorst et al., 2011). Indeed, in primary hepatocytes, the pro-inflammatory cytokine interleukin-1β (IL-1β) is a major inducer of LCN2 (Borkham-Kamphorst et al., 2011). Moreover, we demonstrated that LCN2 has a hepatoprotective effect during liver injury, implying that LCN2 acts to suppress the development and aggravation of liver disease (Borkham-Kamphorst et al., 2011).

Another crucial function of LCN2 is its regulation of energy and lipid metabolism in obese animals. Under metabolic stress conditions such as the feeding of a high fat diet, LCN2 influences the regulation of adipose tissue remodeling (Guo et al., 2016). In line with this finding, LCN2 expression is modified in diabetic animals, and LCN2 levels are significantly higher in the adipose tissues of obese mice and become further upregulated in response to insulin (Wang et al., 2007; Tan et al., 2009). We previously showed that LCN2 is a critical regulator of lipid uptake and stimulates the expression of perilipin 5 (PLIN5/OXPAT), a protein that coats fat droplets (Asimakopoulou et al., 2014). PLIN5 is found surrounding lipid droplets and in the endoplasmic reticulum (ER), mitochondria, and cytosol of hepatocytes. PLIN5 expression is particularly high in oxidative tissues such as skeletal muscle (Greenberg et al., 2011; Koves et al., 2013). PLIN5 maintains the balance between lipogenesis and lipolysis by supporting both fatty acid oxidation and fatty acid storage, and regulates interactions between lipid droplets and mitochondria. Thus, PLIN5 contributes to the metabolic alterations that accompany fasting, overnutrition, and the development of insulin resistance (Dalen et al., 2007; Wang et al., 2011).

At the molecular level, PLIN5 disables lipolysis by either binding to adipose triglyceride synthase (ATGL), which catalyzes the first step of triacylglycerol (TAG) hydrolysis, or by attaching to TAG in lipid droplets (Mason et al., 2014; Wang et al., 2015). Genetic deletion of PLIN5 in mice resulted in a decrease in liver triglycerides (TG) as well as ACC and FAS, proteins promoting fatty acid synthesis (Wang et al., 2015). PLIN5 is also a key predictor of insulin sensitivity and metabolic flexibility (Mason et al., 2014). Accordingly, PLIN5-deficient mice fed a high fat diet show improved glucose tolerance compared to wild type (WT) mice (Mason et al., 2014). This improvement arises because fatty acids derived from intracellular lipolysis are directly converted into sphingolipids, which in turn contribute to insulin resistance (Mason et al., 2014). Thus, both LCN2 and PLIN5 affect energy and fat metabolism in a manner related to insulin sensitivity, and both seem to be pivotal in hepatic steatosis and inflammation because of their hepatoprotective functions.

Overfeeding is one of the main causes of steatosis. If adipose tissues are not able to store lipids any more, free fatty acids (FFA) accumulate in other tissues and organs, mainly as hepatic TG (Firneisz, 2014; Sztalryd and Kimmel, 2014). Triglycerides are also derived from fructose, a highly lipogenic nutrient that is absorbed from the intestine into the portal vein and delivered directly to the liver. Indeed, about 90% of ingested fructose is metabolized in this organ (Softic et al., 2016). High fructose intake induces hepatic lipid accumulation by activating lipogenic gene expression and *de novo* lipogenesis (Basciano et al., 2005; Softic et al., 2016). However, in contrast to glucose, dietary fructose does not stimulate insulin or leptin, which are both important regulators of energy intake. Instead, fructose is a highly efficient activator of the sterol regulatory element-binding transcription factor 1c (SREBP-1c), which regulates genes required for glucose metabolism independently of insulin (Softic et al., 2016).

The metabolism of fructose generates TG that accumulate in the liver, resulting in hepatic insulin resistance and the formation of very low density lipoprotein (VLDL) particles (Basciano et al., 2005; Softic et al., 2016). A factor necessary for the packaging of TG into VLDL is apolipoprotein B-100 (apoB). When hepatic lipids increase, apoB degradation decreases and this factor accumulates in the ER, causing ER stress (Su et al., 2009). In patients suffering from type 2 diabetes, the elevated levels of TG-containing VLDL are toxic because some of these TG are deposited in the muscles, enhancing insulin resistance. Other TGs are deposited in the liver, promoting NASH formation (McGarry, 1992).

An effective method of inducing NAFLD in mice is the feeding of a high fructose diet (Alwahsh et al., 2014; Sodhi et al., 2015). This sugar can mediate the progression of liver disease by inducing dysbiosis in microbiota, leading to inflammatory processes in the intestine, thereby impairing the gut barrier in several ways (Lambertz et al., 2017).

Symptoms include increased insulin resistance, elevated blood pressure, upregulation of markers of oxidative stress, lipogenesis and fibrosis, as well as enhanced expression of LCN2 (Sodhi et al., 2015). To determine the roles of LCN2 and PLIN5 in the formation and progression of steatosis and NAFLD, we examined the liver biology of WT and LCN2-deficient male and female mice subjected to regimens designed to increase their fructose intake over 4 or 8 weeks. We hypothesized high fructose to cause time-dependent liver damage in mice. Because we previously observed that *Lcn2*-deficient mice are more prone to hepatic damage, we expected more severe steatosis in mice lacking LCN2 after prolonged feeding of fructose. In our study, we analyzed male and female mice separately in order to investigate gender-specific differences during progression of disease.

## Materials and methods

### Animals

Female and male mice (8–11 weeks old) were kept one animal to a cage and allowed food and water *ad libitum*. The cages were maintained in a room at constant humidity (50%) and temperature (20°C) with a 12 hr light/dark cycle. Wild type (WT) (*n* = 36) and LCN2 knockout (KO) mice (Berger et al., 2006) (*n* = 30). We used whole-body knockout mice with a gene-targeted *Lcn2* deficiency, in which the targeting vector was designed to replace most of the *Lcn2* coding region including exons 1–5 (Berger et al., 2006). Both genotypes were bred on a C57BL/6 genetic background. Mice were divided into 3 nutritional groups as follows: Control (Co), standard chow and drinking water; FW, standard chow without supplementary sugar (SSNIFF Spezialdiäten GmbH, Soest, Germany) and drinking water supplemented with 30% (w/v) fructose; FF, sugar-free drinking water and a special fructose-containing chow (TD.89247 60% Fructose diet, Envigo Teklad Diets, Madison, WI, USA). Previously such high fructose concentrations were reported to be necessary to induce massive *de novo* lipogenesis and macrosteatosis (DeBosch et al., 2014). These nutritional conditions were maintained for either 4 or 8 weeks. Ten experimental groups were designated as follows: WT/KO Co: WT (*n* = 8) and LCN2 KO mice (*n* = 6) were fed with standard chow; WT/KO FW4: WT (*n* = 8) and LCN2 KO mice (*n* = 6) received fructose in water for 4 weeks; WT/KO FF4: WT (*n* = 6) and LCN2 KO mice (*n* = 6) were fed with fructose-containing chow for 4 weeks; WT/KO FW8: WT (*n* = 8) and LCN2 KO mice (*n* = 6) received fructose in water for 8 weeks; and WT/KO FF8: WT (*n* = 6) and LCN2 KO mice (*n* = 6) were fed with fructose-containing chow for 8 weeks. The total number of 66 animals for this study was approved under number Az.: 84-02.04.2015.A332 by the respective authority which is the *Landesamt für Naturschutz, Umwelt und Verbraucherschutz*, LANUV (https://www.lanuv.nrw.de). Body weights were measured weekly and consumed food and water were calculated every other day by measuring the amounts of food and water given at the beginning of a period and subtracting the amounts consumed after two days. Daily caloric intake was then determined using the following parameters: the fructose-enriched chow provided 3.6 kcal/g food; standard chow provided 3.05 kcal/g food, and the 30% fructose water solution provided 1.188 kcal/ml. At the end of the study, mice were sacrificed by administering an overdose of Isofluran (#B 506, Forene®, Abbott GmbH, Wiesbaden, Germany). All animals used in this study received humane care, and all animal protocols were in full compliance with the guidelines for animal care approved by the German Animal Care Committee.

### Serum analytics

Blood samples were drawn by puncture of the *inferior vena cava* at the time of sacrifice. Blood was collected in 500 μl microcuvettes containing a serum gel (Microvette®, Serum-Gel, Sarstedt, Nürnbrecht, Germany). After blood coagulation, the microvette was centrifuged at 10,000 × g at room temperature (RT) and the serum (supernatant) transferred into an Eppendorf tube and stored at −80°C. Serum samples were analyzed for alanine aminotransferase (ALT), aspartate aminotransferase (AST), glucose, FFA, non-essential fatty acids (NEFA), and TG using standard clinical laboratory tests.

### Liver histology

For hepatic histological analysis, liver tissue was fixed in 4% paraformaldehyde and paraffin-embedded using standard procedures. Paraffin-embedded livers were cut into 5 μm sections and stained with hematoxylin and eosin (H & E) using standard procedures. To assess hepatic steatosis, 10 μm serial cryosections of liver segments were prepared in a cryostat (Leica CM3050 S, Leica, Wetzlar, Germany) at −20°C and stained with Oil Red O. Images were acquired with a mono and colour Nikon light microscope configured with the Eclipse 80i (Nikon, Tokyo, Japan) and equipped with 10 x, 20 x, and 40 x objective lenses.

### Quantitative RT-PCR

Total RNA was isolated from liver tissue using the QIAzol lysis reagent (Qiagen, Hilden, Germany) according to the manufacturer's guidelines, and digested with DNAse using the Purelink RNA Mini kit system (Invitrogen, ThermoFisher, Darmstadt, Germany). Single-stranded cDNA was synthesized from 1 μg purified RNA in a total volume of 20 μl using Superscript II reverse transcriptase and random hexamer primers (all from Invitrogen). Prior to quantitative real-time (qRT)-PCR, cDNA samples were diluted with 80 μl purified water. Aliquots of 5 μl were amplified in a 25 μl volume using SYBR Green™ qPCR SuperMix (Applied Biosystems, Life Technologies, Darmstadt, Germany). Specific primer pairs for mouse mRNA amplification were designed using the Universal Probe Library (UPL) tool from Roche Diagnostics (Mannheim, Germany). After initial denaturation at 95°C for 10 min, DNA was amplified using 40 cycles at 95°C for 15 s and 60°C for 1 min. All PCR reactions were performed in duplicate and normalized to the housekeeping gene glyceraldehyde 3-phosphate dehydrogenase (GAPDH). Relative levels of target mRNAs were calculated using the 2^−ΔΔCT^ method (Livak and Schmittgen, 2001), and relative mRNA expression levels were expressed as the normalized quantity of target mRNA relative to the normalized quantity of control mRNA. All primers used in qRT-PCR are given in Table 1.

**Table 1:** Primers used for qRT-PCR.

**Gene**	**Access. no**.	**Primer (5′ → 3′)**
Accα	XM_006531957.2	For: acagagatggtggctgatgtc Rev: gatccccatggcaatctg
Accβ	XM_006530111.2	For: ctccgtgctatgccagttc Rev: ttcaggagctcgggatgtag
CPT1	NM_153679	For: agagaagcctgccagtttgtgaga Rev: tgtacagtgcaaagaggtgacggt
DGAT1	NM_010046	For: tcgtggtatcctgaattggtg Rev: aggttctctaaaaataaccttgcatt
DGAT2	AF384160	For: gctggtgccctactccaag Rev: ccagcttggggacagtga
FAS	NM_007987.2	For: tgcagacatgctgtggatct Rev: cttaactgtgagccagcaagc
GAPDH	XM_001473623	For: actgccacccagaagactg Rev: caccaccctgttgctgtag
HSL	NM_010719.5	For: agcgctggaggagtgtttt Rev: ccgctctccagttgaacc
LCN2	NM_008491.1	For: ccatctatgagctacaagagaacaat Rev: tctgatccagtagcgacagc
LXRα	AF085745.1	For: cgcgacagttttggtagagg Rev: ctccagccacaaggacatc
PLIN5	NM_025874.3	For: gtcggagaagctggtggac Rev: tcagctgccaggactgcta
Scd1	NM_009127.4	For: ttccctcctgcaagctctac Rev: cagagcgctggtcatgtagt
SREBP1c	AF374266.1	For: acaagattgtggagctcaaagac Rev: gcgcaagacagcagatttatt

### Western blot analysis

Liver tissues were homogenized in RIPA lysis and extraction buffer containing 20 mM Tris-HCl (pH 7.2), 150 mM NaCl, 2% (w/v) NP-40, 0.1% (w/v) SDS, 0.5% (w/v) sodium deoxycholate, and the Complete™-mixture of proteinase inhibitors (Roche). After measuring the protein concentration of each extract, equal protein amounts obtained from mice belonging to the same group were pooled, and 60 μg total protein per lane was mixed with NuPAGE™LDS electrophoresis sample buffer containing dithiothreitol (DTT). Samples were heated at 80°C for 7 min and separated in 4–12% Bis-Tris gels in MES [2-(N-morpholino)ethanesulfonic acid] running buffer (Invitrogen). Proteins were transferred to a Protran membrane (GE Healthcare Life Science, Freiburg, Germany) and equal protein loading was confirmed by Ponceau S staining. Membranes were blocked for 1 hr at RT in 5% (w/v) non-fat milk solution in Tris-buffered saline (TBST) supplemented with 0.1% Tween-20 (Carl ROTH, Karlsruhe, Germany). Blocked membranes were incubated overnight at 4°C with primary antibodies diluted in 2.5% (w/v) non-fat milk powder in TBST. After washing, membranes were incubated for 2 h at RT with HRP-conjugated secondary IgG antibodies (Santa Cruz Biotech, Santa Cruz, CA) directed against mouse, rabbit, and goat. Detection was performed using the SuperSignal chemiluminescent substrate (Pierce, Bonn, Germany). All antibodies used in this study are listed in Table 2.

**Table 2:** Antibodies used in this study.

**Antibody**	**Cat. No**	**Clonality**	**Supplier**	**Species reactivity**	**Dilution**
**PRIMARY ANTIBODIES**					
β-Actin	A5441	Monoclonal mouse IgG1	Sigma-Aldrich, Taufkirchen, Germany	human, mouse, rat	1:5,000
FABP5	AF1476	Polyclonal goat IgG	R&D Systems, Wiesbaden, Germany	human, mouse, rat	1:1,000
LCN2/NGAL	AF3508	Polyclonal goat IgG	R&D Systems	mouse, rat	1:1,000
PLIN5/OXPAT	NB-110-60509	Polyclonal rabbit	Novus Biologicals, Wiesbaden, Germany	human, mouse	1:1,000
**SECONDARY ANTIBODIES**					
IgG-HRP	sc-2004	Goat anti-rabbit IgG	Santa Cruz, Santa Cruz, CA, USA	rabbit	1:5,000
IgG-HRP	sc-2005	Goat-anti-mouse IgG	Santa Cruz	mouse	1:5,000
IgG-HRP	sc-2056	Donkey-anti-goat IgG	Santa Cruz	goat	1:5,000

### Statistical analysis and software

All values are reported as the mean plus standard deviation as constructed with Microsoft Excel (Microsoft Corporation, Redmond, WA, USA). Statistically significant differences between fructose-treated and control groups were assessed by one-way ANOVA using GraphPad Prism software version 6 (GraphPad Software, Inc., La Jolla, CA, USA). This test was used to assess the differences between treatments. *p* < 0.05 were considered to be statistically significant. All significant p values, F ratios, and *R*^2^ are listed in Table 3. Male and female mice were analysed separately. In order to distinguish if the observed effects were due to the different genotypes or result of fructose treatment, we examined the data in three different sample compilations. Firstly, we compared all data appointing WT Co as the control group and eight treatment groups, leaving out KO Co (ANOVA all). Significance is depicted as a line starting at WT Co and ending at KO FF8 which is the last treatment group used in this part of statistical evaluation. Secondly, we used the WT mice only in the statistic analysis in order to get a closer view on the fructose treatment on WT mice. WT Co was used as a control group comparing with four different treatment groups, leaving out all *Lcn2* KO mice (ANOVA WT). In this case, the significances are showed with a line starting at WT Co ending up at WT FF8 as the last treatment group. Thirdly, we focused only on the KO mice using KO Co as a control group and the other KO groups as treatment groups without accounting the WT mice (ANOVA KO). Significances are represented with a line starting at KO Co and ending at KO FF8 as the last treatment group. Statistical significances are indicated as ^*^ < 0.05, ^**^ < 0.01, ^***^ < 0.001, and ^****^ < 0.0001, respectively.

**Table 3:** One-way ANOVA significances.

**Statistics to**	**Analyte**	**Gender**	**ANOVA (p-value)**			**ANOVA (F-ratio)**			**ANOVA (*****R***^**2**^**)**		
			**all**	**WT**	**KO**	**all**	**WT**	**KO**	**all**	**WT**	**KO**
Liver weight (Figure 2)	rel. liver weight	♀	0.0322	NS	NS	2.74	1.998	1.786	0.5229	0.3807	0.4425
		♂	0.0002	NS	NS	6.473	2.086	1.083	0.7018	0.3909	0.2825
Serum analytics (Figures 4, 5)	AST	♀	NS	0.0226	NS	2.008	4.124	0.552	0.4582	0.5593	0.2163
		♂	NS	NS	NS	0.8302	0.2274	0.5873	0.2319	0.06539	0.1902
	ALT	♀	NS	0.0239	NS	1.332	4.054	0.1997	0.3594	0.555	0.09081
		♂	NS	NS	NS	0.8302	0.6673	0.4479	0.2319	0.1703	0.1519
	Glucose	♀	NS	0.0149	NS	1.197	4.831	0.1889	0.3473	0.6169	0.08632
		♂	0.0206	0.0073	NS	2.965	5.658	1.518	0.5188	0.6351	0.3778
	Triglycerides	♀	NS	NS	NS	1.796	2.221	1.718	0.4306	0.406	0.4621
		♂	NS	NS	NS	0.9294	0.9925	0.9956	0.2526	0.2339	0.2848
	FFA/NEFA	♀	NS	NS	NS	1.064	1.05	1.692	0.3176	0.2592	0.4582
		♂	0.0001	0.0009	0.0129	8.252	10.73	6.413	0.7857	0.796	0.7623
*Lcn2* and *Oxpat* expression (Figure 6)	*Lcn2*	♀	0.0003	NS	NS	6.37	1.351	0.4255	0.7414	0.3294	0.159
		♂	0.0003	NS	NS	6.37	1.351	0.4255	0.7414	0.3294	0.159
	*Plin5/Oxpat*	♀	0.0448	NS	NS	2.587	3.249	0.6958	0.5348	0.5416	0.2362
		♂	NS	NS	NS	1.002	1.02	0.6254	0.2907	0.2705	0.1939
Fatty acid synthesis and oxidation (Supplemental Figure 2)	SREBP-1c	♀	0.0383	NS	0.0172	2.658	2.408	5.375	0.5282	0.4453	0.7049
		♂	0.0288	NS	0.0196	2.779	1.498	4.622	0.5142	0.3331	0.627
	Accα	♀	NS	NS	0.0316	1.917	2.083	4.334	0.46	0.431	0.6583
		♂	0.0273	NS	0.0055	2.815	3.23	6.718	0.5175	0.5185	0.7095
	Accβ	♀	NS	NS	NS	0.8263	1.239	2.108	0.2686	0.3105	0.4837
		♂	0.0422	NS	NS	2.529	3.237	0.7876	0.4907	0.519	0.2226
	FAS	♀	0.0447	NS	NS	2.588	2.413	2.862	0.5349	0.4674	0.5598
		♂	NS	NS	NS	0.5272	0.5016	0.8447	0.1673	0.1432	0.235
	Scd1	♀	NS	0.0449	NS	1.323	3.386	1.243	0.3577	0.5302	0.3559
		♂	NS	NS	NS	0.3833	0.2937	0.1868	0.1274	0.08918	0.0636
	DGAT1	♀	NS	NS	NS	0.7929	0.7463	2.016	0.2606	0.2135	0.4726
		♂	NS	NS	NS	2.137	2.057	3.116	0.4488	0.4067	0.5312
	DGAT2	♀	NS	NS	NS	1.1	1.456	0.8437	0.3284	0.3462	0.2727
		♂	NS	NS	NS	1.367	1.455	0.6702	0.3424	0.3266	0.1959
	CPT1	♀	NS	NS	NS	2.447	2.828	0.9074	0.5502	0.5569	0.2874
		♂	NS	NS	NS	2.394	2.042	1.65	0.5448	0.5052	0.3976
	HSL	♀	NS	NS	NS	1.007	0.9478	0.6465	0.335	0.2964	0.2232
		♂	0.0072	0.045	NS	4.067	4.01	1.393	0.6568	0.6672	0.3362
	LXRα	♀	NS	NS	NS	1.309	1.206	3.429	0.3553	0.2867	0.6038
		♂	NS	NS	NS	0.7983	1.021	0.4134	0.242	0.2707	0.1307

*Accα/β, Acetyl-CoA carboxylase α or β; ALT, alanine aminotransferase; AST, aspartate-aminotransferase; CPT1, carnitine palmitoyltransferase 1; DGAT1/2, diacylglycerol acyltransferase1 or 2; FAS, fatty acid synthase; FFA, free fatty acid(s); HSL, hormone-sensitive lipase; KO, knockout; Lcn2, lipocalin 2; LXRα, liver X receptor α; NEFA, non-essential fatty acid(s); NS, not significant; Oxpat/Plin5, perilipin 5; Scd1, stearoyl-CoA desaturase 1; SREBP-1c, sterol regulatory element-binding transcription factor 1c; WT, wild type*.

## Results

### Excess fructose induces fatty liver disease in *Lcn2* deficient mice

**Figure 1:**
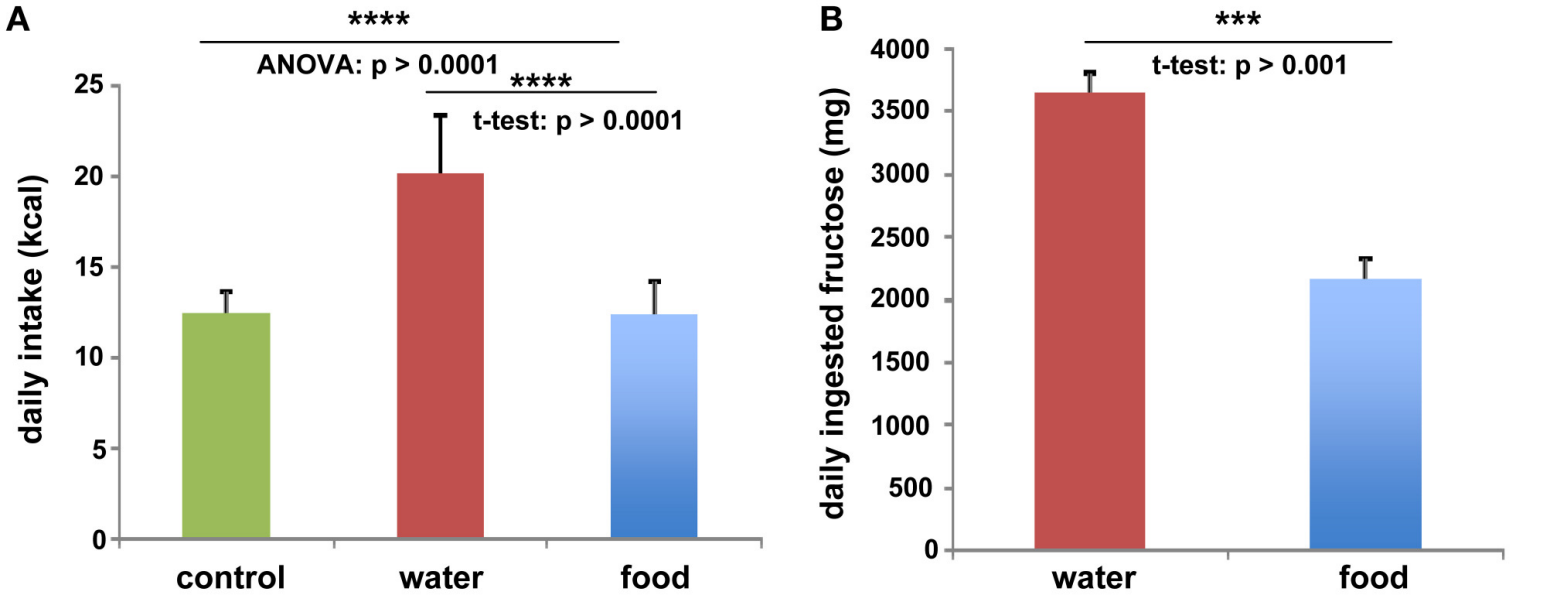
Fructose and energy balance. Averaged values for **(A)** daily kcal and **(B)** fructose intake from all animals, irrespectively of feeding times. Differences reaching significance are marked by asterisks (^***^p > 0.001, ^****^p > 0.0001).

**Figure 2:**
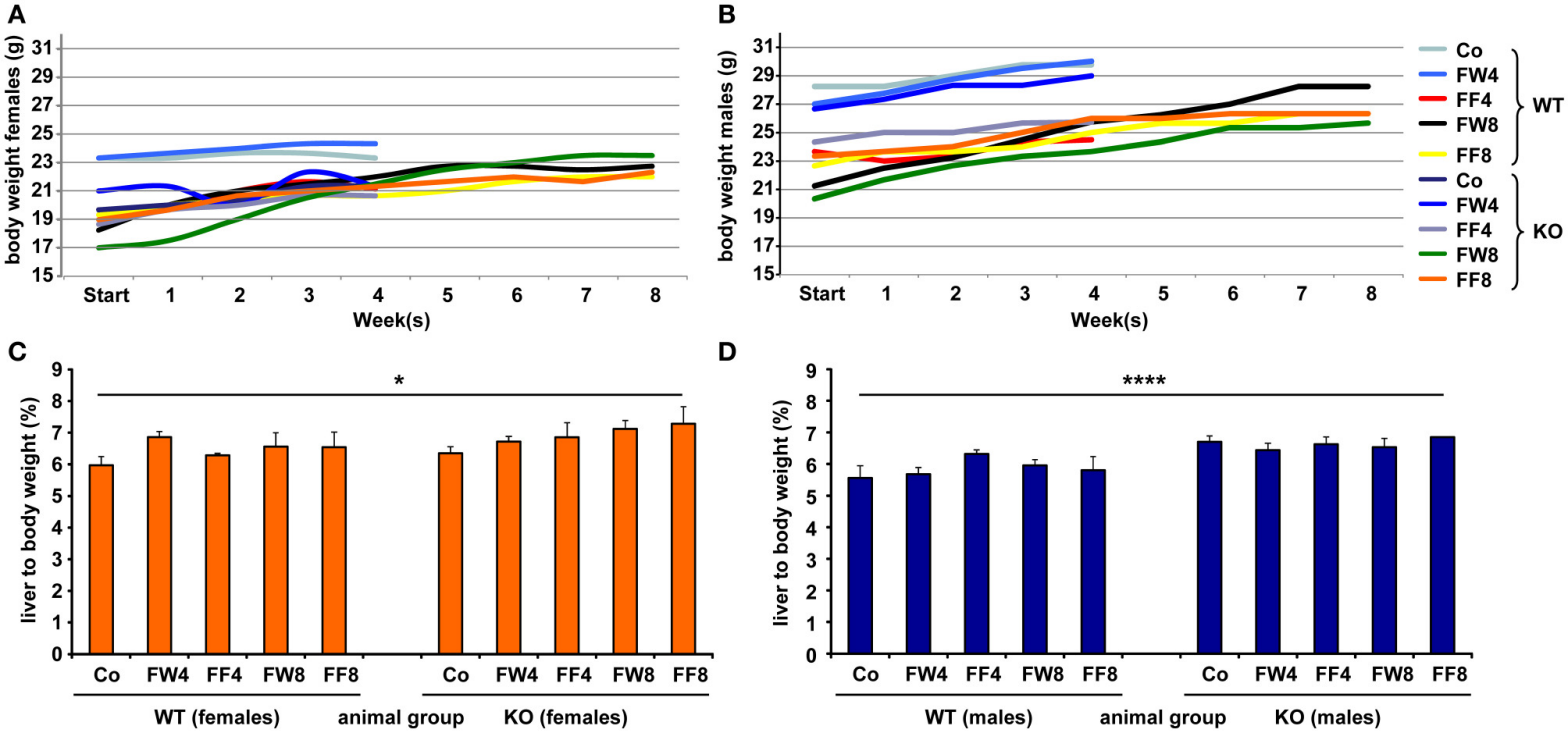
Comparative analysis of body and liver to body weights. Development of body weights of **(A)** female and **(B)** male mice fed with the indicated diets. At the different time points, the mean body weight of control males was higher than that of control females. Relative liver to body weights of **(C)** female and **(D)** male mice fed with standard or fructose-enriched diets. Significant differences between groups as determined by ANOVA testing are indicated by asterisks (^*^p > 0.05, ^****^p > 0.0001).

To investigate the function of LCN2 and PLIN5 in the formation and progression of NAFLD, we fed WT and LCN2 KO mice with two different high-fructose diets for 4 or 8 weeks. Fructose was administered either via the animals' food, or via a 30% (v/v) solution in their drinking water (Supplemental Figure 1). Animals that received standard chow and regular drinking water served as controls. The daily caloric intake in control mice was similar to that of animals that received the FF diet, while the daily ingested fructose and energy intake were significant higher in animals receiving fructose via the drinking water (Figures 1A,B). Both sexes reduced their food intake after receiving solid food enriched in fructose (data not shown). Moreover, the body weights of male mice increased more rapidly than in female mice, independently if fructose was given in food or drinking water (Figures 2A,B and Table 4). In addition, male and female LCN2 KO mice exhibited significantly higher liver and higher liver to body weights compared to WT controls that was more pronounced males than in females (ANOVA all: *p* = 0.0271 females, *p* = 0.0001 males) (Figures 2C,D, Tables 3, 5), suggesting that females might be more susceptible to liver damage when exposed to fructose. Thus, fructose treatment does not cause any significant differences between WT and *Lcn2*-deficient mice, but, in general, null mice showed a higher liver weight.

**Table 4:** Body and liver weight.

**Group**		**Body weight (g)**	**Liver weight (g)**	**Rel. liver weight (%)**
WT (♀)	Control	22.64	1.43	6.32
		23.46	1.43	6.10
		23.13	1.28	5.53
		23.18	1.37	5.90
	FW4	22.85	1.57	6.87
		22.91	1.49	6.50
		24.45	1.66	6.79
		24.66	1.59	6.44
	FF4	20.83	1.29	6.16
		20.50	1.30	6.34
		21.90	1.37	6.25
	FW8	22.02	1.58	7.17
		21.59	1.46	6.76
		23.40	1.47	6.30
		21.31	1.28	6.00
	FF8	20.11	1.31	6.51
		20.15	1.44	7.15
		24.00	1.43	5.96
LCN2 KO (♀)	Control	21.04	1.36	6.46
		20.0	1.21	6.05
		19.91	1.30	6.53
	FW4	23.00	1.52	6.60
		24.00	1.58	6.58
		20.81	1.45	6.96
	FF4	18.17	1.31	7.21
		20.83	1.29	6.19
		22.50	1.60	7.15
	FW8	24.75	1.83	7.39
		22.04	1.51	6.85
	FF8	24.30	1.54	6.34
		20.83	1.66	7.97
		21.71	1.57	7.23
WT (♂)	Control	29.00	1.70	5.86
		30.00	1.63	5.43
		30.00	1.51	5.03
		30.00	1.81	6.03
	FW4	28.00	1.53	5.50
		32.00	1.91	6.00
		29.0	1.71	5.90
		30.00	1.81	6.03
	FF4	22.68	1.47	6.48
		24.45	1.30	6.31
		26.06	1.61	6.18
	FW8	28.58	1.78	6.22
		26.95	1.56	5.79
		27.92	1.69	6.05
		26.88	1.55	5.77
	FF8	25.85	1.45	5.60
		23.62	1.51	6.40
		25.45	1.38	5.42
LCN2 KO (♂)	Control	26.75	1.83	6.84
		25.10	1.76	6.83
		25.78	1.74	6.45
	FW4	29.00	1.93	6.66
		28.00	1.78	6.14
		30.00	1.96	6.53
	FF4	26.18	1.68	6.42
		25.12	1.64	6.53
		26.49	1.84	6.94
	FW8	25.67	1.79	6.97
		25.17	1.66	6.56
		25.80	1.61	6.24
		25.11	1.60	6.37
	FF8	26.82	1.83	6.82
		24.46	1.68	6.67
		25.78	1.82	7.07

*FF4, Fructose in food fed for 4 weeks; FF8, fructose in food fed for 8 weeks; FW4, fructose in water fed for 4 weeks; FW8, fructose in water fed for 8 weeks; LCN2 KO, Lipocalin 2 knockout; WT, wild type*.

**Table 5:** Up- or down-regulation of genes after fructose treatment.

**Gene**	**Gender**	**WT**	**KO**	**ANOVA all (*p-*value)**	**ANOVA WT (*p*-value)**	**ANOVA KO (*p-*value)**
LCN2	♀	+	0	0.0003	NS	NS
	♂	+	0	0.0003	NS	NS
Oxpat/Plin5	♀	−	−	0.0448	NS	NS
	♂	0	0	NS	NS	NS
SREBP-1c	♀	+ (FF)	↑ (FF)	0.0383	NS	0.0172
	♂	+ (FF)	↑ (FF)	0.0288	NS	0.0196
Accα	♀	+	↑	NS	NS	0.0316
	♂	+	↑	0.0273	NS	0.0055
Accβ	♀	+	+	NS	NS	NS
	♂	+	+	0.0422	NS	NS
FAS	♀	−	−	NS	NS	NS
	♂	−	−	NS	NS	NS
Scd1	♀	−	−	NS	0.0449	NS
	♂	0	0	NS	NS	NS
DGAT1	♀	−	0	NS	NS	NS
	♂	+	0	NS	NS	NS
DGAT2	♀	0	0	NS	NS	NS
	♂	+	0	NS	NS	NS
CPT1	♀	+	0	NS	NS	NS
	♂	−	−	NS	NS	NS
HSL	♀	0	0	NS	NS	NS
	♂	↓	−	0.0072	0.045	NS
LXRα	♀	−	−	NS	NS	NS
	♂	0	0	NS	NS	NS

*LCN2, Lipocalin 2; Oxpat/Plin5, perlipin 5; SREBP-1c, sterol regulatory element-binding protein-1c; Accα or β, acetyl-CoA carboxylase α or β; FAS, fatty acid synthase; Scd1, stearoyl-CoA desaturase 1; DGAT1 or 2, diacylglycerol acyltransferase1 or 2; CPT1, carnitine palmitoyltransferase 1; HSL, hormone-sensitive lipase; LXRα, liver X receptor α; ♀, female mice; ♂, male mice; +, trend to increase during fructose feeding; 0 = no changes during fructose feeding; -, trend to decrease during fructose feeding; ↑, significant increase during fructose feeding; ↓, significant decrease during fructose feeding; (FF), differences only noticed during fructose feeding in food; NS, not significant*.

### Excess fructose induces hepatic steatosis

**Figure 3:**
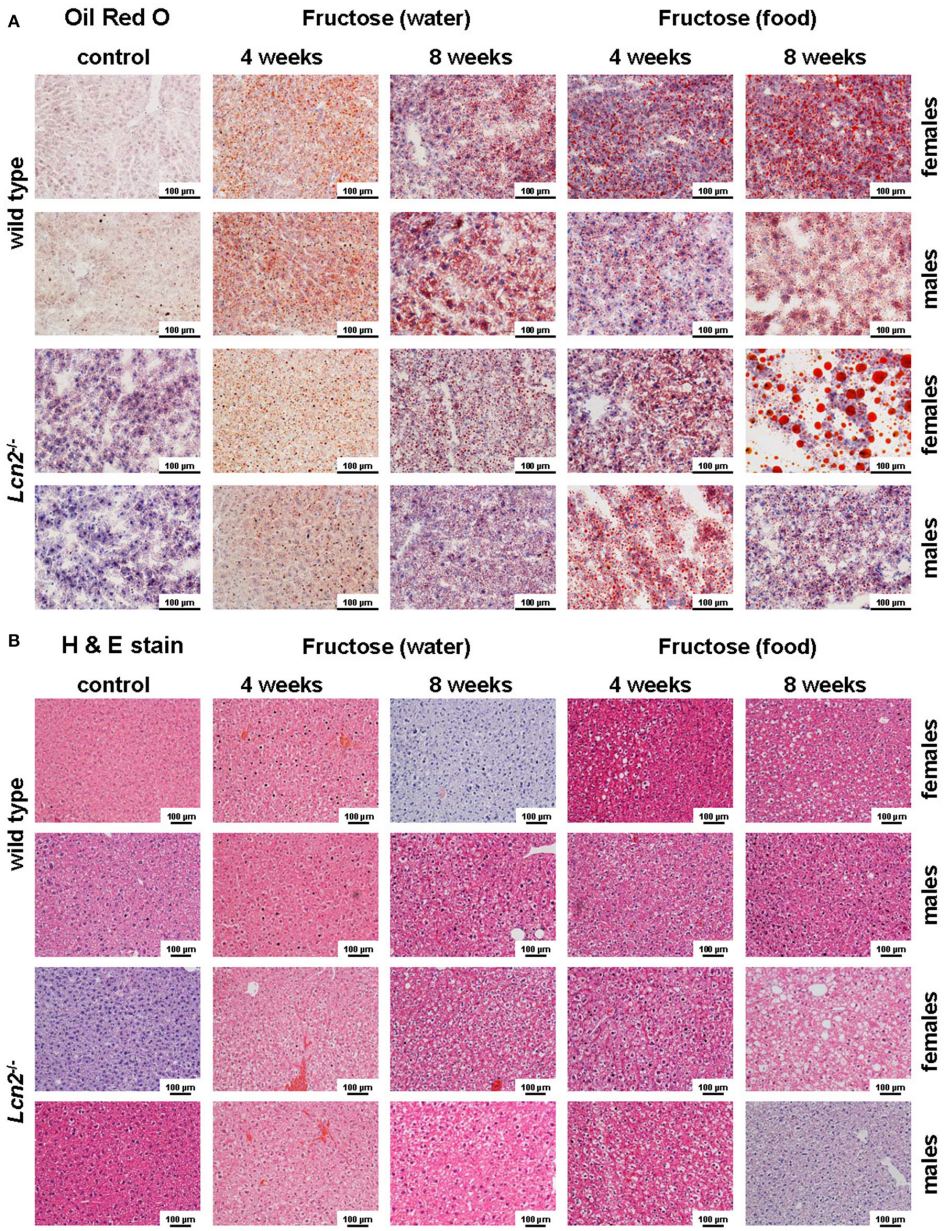
Oil Red O and H&E staining. **(A)** Hepatic fat content was visualized by Oil Red O staining. Fructose administered in either food or water caused fatty liver disease. Female mice showed more severe fat accumulation in the liver than male mice, and LCN2 KO mice exhibited more fat deposits than WT mice. Bars, 100 μm. **(B)** H & E-stained liver sections of the indicated groups of control and fructose-treated mice. Bars, 100 μm.

Oil Red O staining revealed hepatic fat accumulation in fructose-treated mice that progressed markedly from 4 to 8 weeks (Figure 3A). Furthermore, fructose feeding resulted in more hepatic fat deposits in female mice compared to males. In line with previous studies (Bachman et al., 2009; Borkham-Kamphorst et al., 2011; Asimakopoulou et al., 2014), male LCN2 KO mice showed greater hepatic damage than WT mice after the hepatotoxic challenge represented by prolonged fructose feeding. This increased hepatic damage in WT females and all mice lacking LCN2 was also documented by H & E staining (Figure 3B).

### A lack of LCN2 exacerbates hepatic and metabolomic damage induced by excess fructose

**Figure 4:**
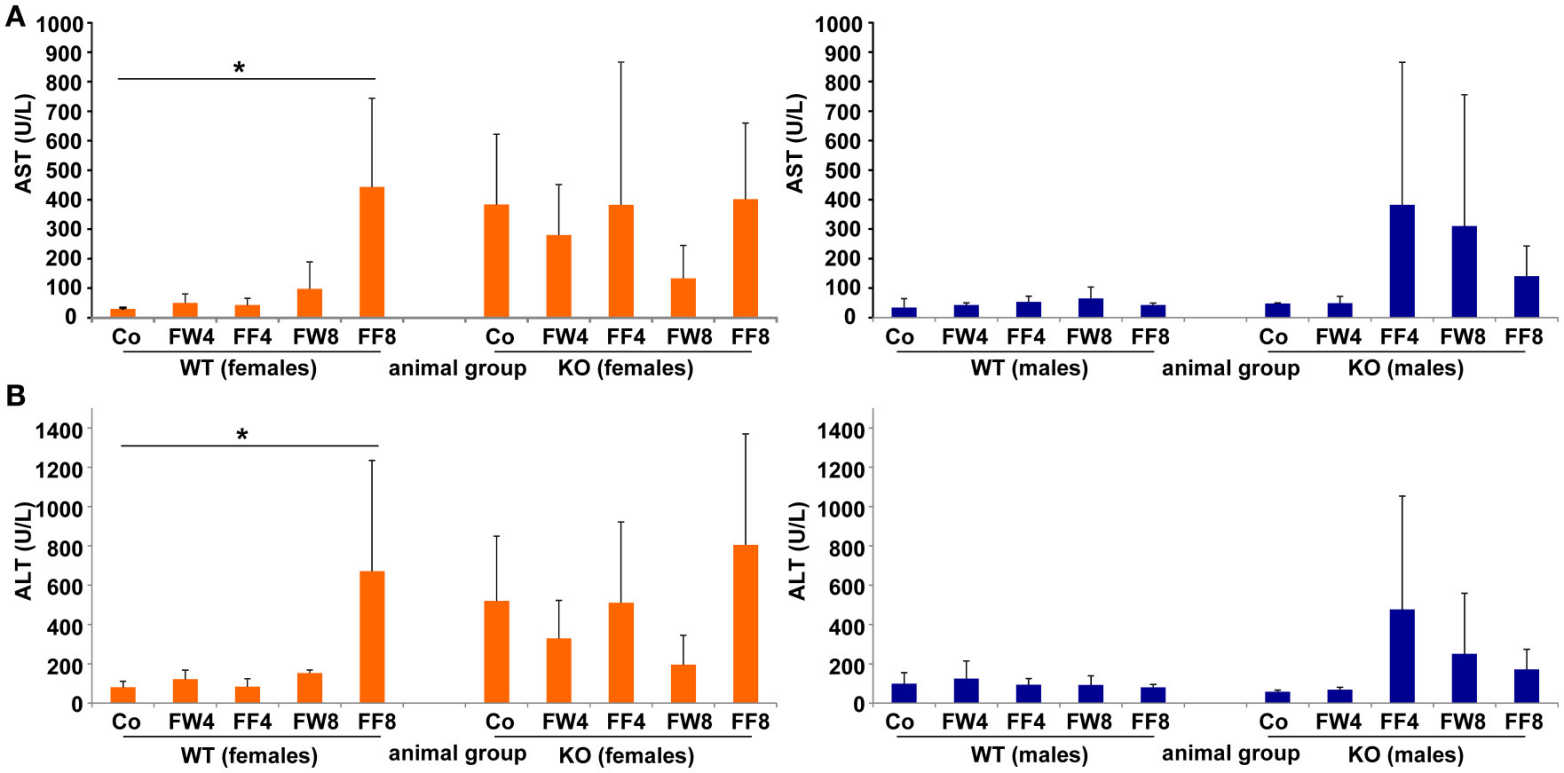
Serum levels of AST, ALT. **(A)** Serum AST and **(B)** ALT activities were measured in the indicated mouse groups using standard tests. Significant values observed were: ^*^p > 0.05.

Elevated levels of the liver enzymes AST and ALT are biomarkers of liver damage. Although the serum values of ALT and AST across all experimental groups appeared quite similar, WT females showed significanct differences between the treatment groups in these parameters (ANOVA WT: AST, *p* = 0.0226; ALT, *p* = 0.0239) (Table 3), especially in female mice fed with fructose for 8 weeks (FF8) (Figures 4A,B). In contrast, AST and ALT values in LCN2 KO mice were generally higher compared to those in WT mice but did not reached significant differences within the treatment groups.

**Figure 5:**
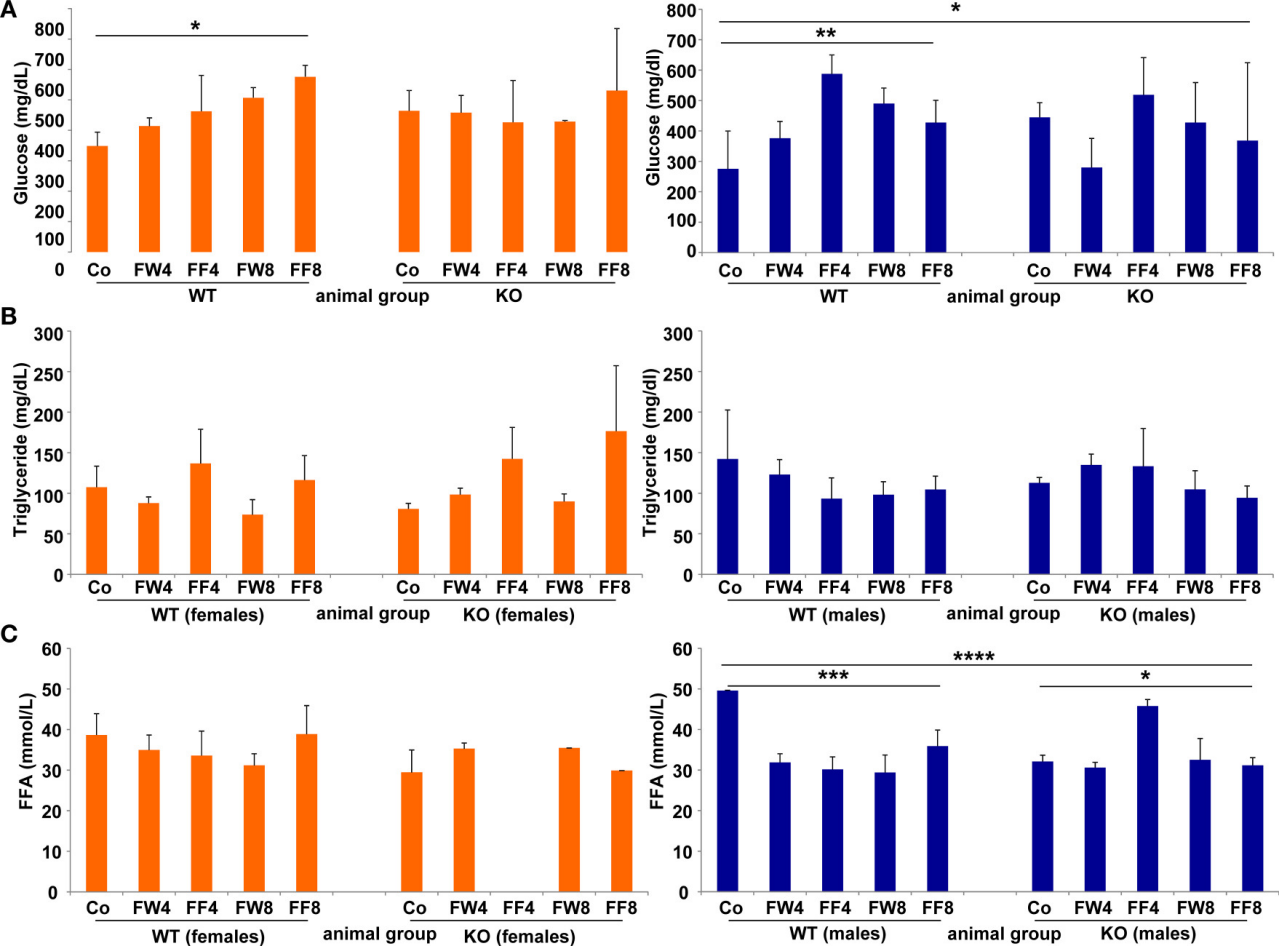
Glucose, triglycerides, and free fatty acids. Serum concentrations of **(A)** glucose, **(B)** TG, and **(C)** free fatty acids were measured without fasting before sample collection. The results are gender-specifically depicted (female: left panels; male: right panels). Significant differences between groups as determined by ANOVA testing are indicated by asterisks (^*^p > 0.05, ^**^ *p*> 0.01, ^***^p > 0.001, and ^****^p > 0.0001).

WT females showed significant differences in serum glucose in response to high fructose feeding (Figure 5A) (ANOVA WT: *p* = 0.0149), with a clear trend in rising serum glucose during treatment, an event not observed in female mice lacking LCN2. Similar results were noted in male mice (ANOVA WT: *p* = 0.0073). The consumption of large amounts of sugars triggers the production of fatty acids in order to store energy. Fructose is a sugar particularly capable of stimulating lipogenic pathways, propagating production of fatty acids and TG (Basciano et al., 2005). In our study, serum TG levels were not altered by either fructose treatment or loss of LCN2 (Figure 5B). Concentrations of serum FFA were highly variable among all groups, with more variation among males than females (ANOVA all: *p* = 0.0001) (Figure 5C). Both, the WT and KO males, exhibited significant distinction considering the genotypes (ANOVA WT: *p* = 0.0009, ANOVA KO: *p* = 0.0129).

### Fructose consumption alters gene expression patterns

Table 5 summarizes genes involved in fat metabolism that became up- or downregulated during fructose treatment. Student *t*-test analysis comparing non-treated WT and LCN2 KO showed no significant differences (data not shown).

**Figure 6:**
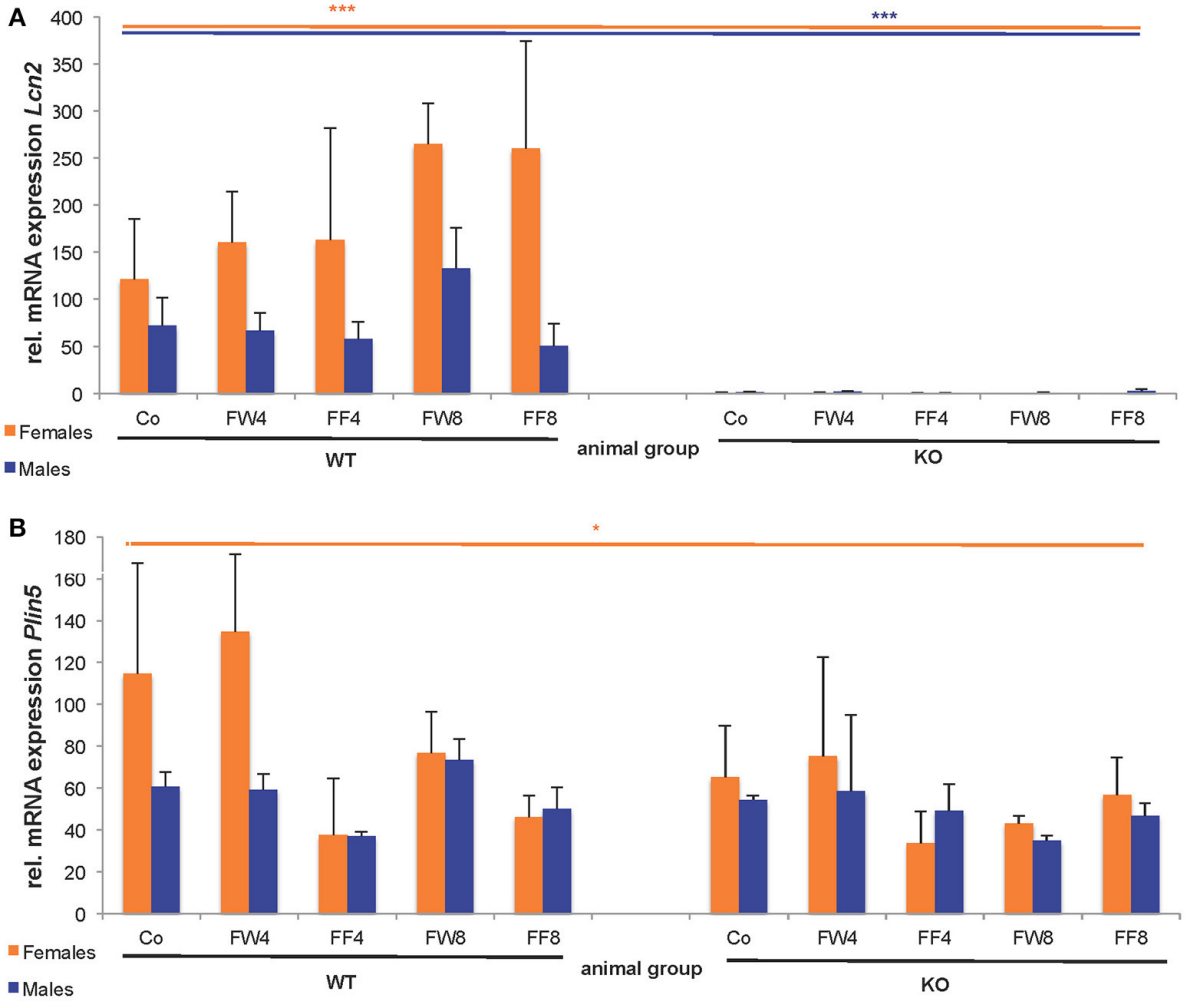
Hepatic expression of LCN2 and PLIN5/OXPAT. Relative mRNA levels of **(A)** *Lcn2* and **(B)** *Plin5/Oxpat* were measured by qRT-PCR. Expression values were normalized to GAPDH content. Significant differences between groups as determined by ANOVA testing are ^*^p > 0.05 and ^***^p > 0.001.

In WT female mice, the relative mRNA expression of Lcn2 varied significantly between the treatment groups, irrespective if fructose was added to the drinking water or to the chow (Figure 6A). This induction of LCN2 was not observed in male mice. As expected, LCN2 KO mice did not express measurable LCN2 mRNA. In male mice, there were no differences among groups in *Plin5/Oxpat* mRNA expression (Figure 6B). In contrast, fructose-treated females showed significant differences within the groups (ANOVA all: p = 0.0448) and a marked downregulation in *Plin5/Oxpat* during fructose treatment.

In our experimental set-up, SREBP-1c mRNA levels were significantly altered in female FF groups (Supplemental Figure 2, Table 5). In the treatment groups of both, WT and LCN2 KO mice, a significant difference in hepatic SREBP-1c expression was observed (s all: *p* = 0.0383 female, *p* = 0.0288 males). In addition, SREBP-1c expression was also significantly different in livers of LCN2 KO mice compared to those of WT mice (ANOVA KO: *p* = 0.0172 females, *p* = 0.0196 males) (Tables 3, 5).

In both WT and LCN2 KO mice, as well as in female and male mice, acetyl-CoA carboxylase α (ACCα) tended to be upregulated by fructose consumption (Supplemental Figure 2). However, this effect reached significance only in the LCN2 KO mice (ANOVA KO: *p* = 0.0316 females, *p* = 0.0055 males). In line with this finding, the difference between the genotypes was altered significantly when sex was considered (ANOVA all: *p* = 0.045 males). The expression of ACCβ mRNA also tended to increase during fructose ingestion (Table 5). A significant difference comparing all groups was detected in males (ANOVA all: *p* = 0.0422).

However, unexpectedly, we found that FAS mRNA was suppressed in fructose-fed female mice considering both genotypes (Supplemental Figure 2, Table 5). LCN2 KO female mice on a control diet expressed less FAS mRNA than female WT controls Nevertheless, a significant difference between WT and LCN2 KO mice was seen only in females (ANOVA all: *p* = 0.0447) and not in males, and not among experimental groups. These data suggest that fructose intake had no significant influence on FAS mRNA expression in this study.

We observed that, in WT female mice, the relative mRNA expression of *Scd1* was upregulated during early phases of fructose feeding, but dropped to the baseline during longer feeding periods (ANOVA WT: *p* = 0.0449). Male mice showed no upregulation of *Scd1*. In contrast, mRNA levels of diacylglycerol acyltransferase1 (DGAT1) and 2 (DGAT2), which catalyze TG formation from diacylglycerides, appeared to be upregulated in WT male mice fed fructose for 8 weeks. This increase was suppressed in male LCN2 KO mice and not found in females.

We next tested if fructose treatment influenced the expression of lipolytic marker genes. None of the mice showed differences in *CPT1* mRNA after fructose consumption.

Hormone-sensitive lipase (HSL) is involved in the hydrolysis of intracellular TG stores. HSL levels were not affected by fructose feeding in female mice but males showed a difference between the genotypes (ANOVA all: *p* = 0.0072) (Tables 3, 5). HSL was depressed in fructose-fed WT mice (ANOVA WT: *p* = 0.045). These results indicate that fructose consumption has an impact on HSL expression in WT male mice but less in the absence of LCN2.

### No effect of fructose consumption or LCN2 loss on Lxrα

Liver X receptor α (LXRα) plays an important role in the transcriptional control of lipid homeostasis and inflammation. We found, using qRT-PCR analysis, that LXRα expression was not affected by fructose treatment or disruption of the LCN2 gene in our experimental setting.

### Females and LCN2-deficient mice exhibit more fat in the liver after fructose exposure

**Figure 7:**
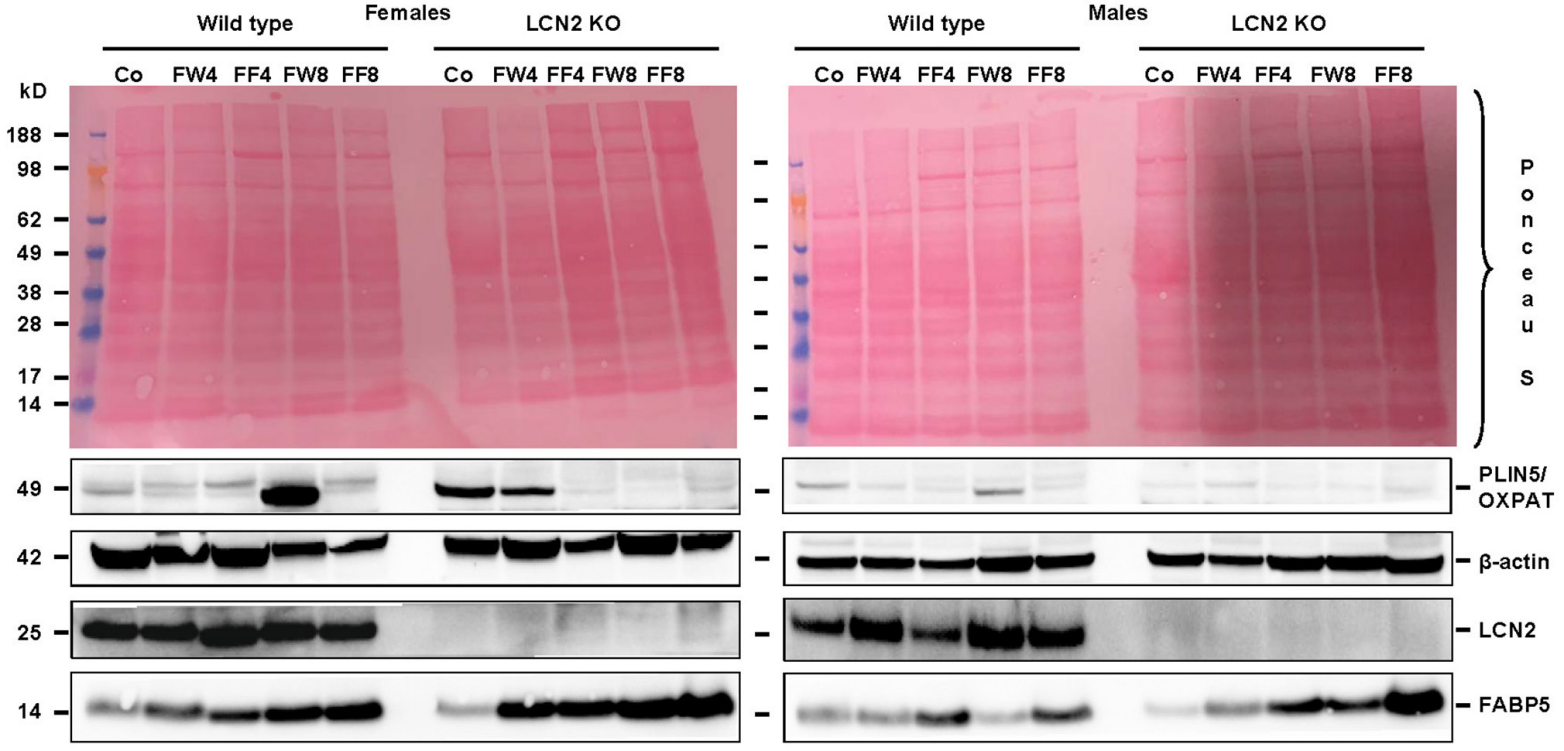
Western blot analysis. Liver extracts from female **(left)** or male **(right)** mice were analyzed by Western blot analysis for expression of PLIN5/OXPAT, β-actin, LCN2, and FABP5. Ponceau S stains served as further controls for equal protein loading. Fructose feeding enhanced FABP5 expression in a time-dependent manner. The effect was more prominent in females than in males and in *Lcn2*-deficient mice than in WT mice.

Examination of our experimental groups revealed that fructose treatment enhances the expression of Fatty acid binding Protein 5 (FABP5) in a time-dependent manner in both WT and LCN2 KO mice, and upregulates this protein more in females than in males (Figure 7). In addition, LCN2-deficient mice expressed more FABP5 than did WT mice. Confirming our PCR data, females on the control diet showed more LCN2 expression than WT males on this diet, and express also more LCN2 when exposed to a fructose diet.

## Discussion

### Fructose induces steatosis and liver damage

We previously reported that LCN2-deficient mice exhibit more hepatic fat than WT controls regardless of their diet, indicating that LCN2 has a major influence on lipid homeostasis and lipid droplet formation (Asimakopoulou et al., 2014). Furthermore, LCN2 is an established marker of liver damage and homeostasis since it has an important role in hepatic inflammatory responses (Borkham-Kamphorst et al., 2011).

In the current study, we chose two different high-fructose diets to induce liver damage in mice. To investigate if the route of fructose intake affected the severity of liver damage, we compared the effects of feeding mice with chow containing 60% fructose with those of supplying the mice with drinking water containing 30% fructose.

Liver weight differed with regard to genotype, but fructose feeding didn't cause a significant increase in liver weight in either sex (Figures 2C,D). However, independent of diet, mice lacking LCN2 generally had greater liver weights, confirming our previous results showing that loss of LCN2 results in hepatic fat accumulation (Asimakopoulou et al., 2014). Although our LCN2-deficient mice were prone to developing higher liver weights during fructose exposure, similarly-treated control mice also showed higher liver weights, suggesting that fructose alone has an influence on liver weight, and that the hepatotoxic effects induced by fructose are elevated in the absence of LCN2.

Surprisingly, relative liver weights were not increased in WT mice during prolonged fructose treatment, unlike in LCN2 KO mice whose liver weights increased continuously (Figures 2C,D). These data suggest that fructose feeding had only a minor effect on liver weight in WT mice but stimulated considerable weight gain in the lives of mice lacking LCN2. Although females also exhibited higher liver weights in relation to their body weight, the significance of this gain was more pronounced in males than in females. Thus, an absence of LCN2 in male mice has a greater impact on liver weight than in females, which have higher liver weights in general.

Our analysis of liver tissues by Western blot suggested an increased susceptibility to steatosis conferred by LCN2 deficiency (Figure 7). LCN2 KO mice exhibited much more FABP5 when treated with fructose in comparison to WT mice, and females again possessed higher expression of this protein compared to males, suggesting a higher fat content in livers of females. Certainly, our Western blot data indicate a critical influence of fructose exposure on hepatic fat content. It is tempting to speculate that WT mice exposed to fructose for a longer period would be more affected by this sugar, and that a deficiency in LCN2 confers greater susceptibility to steatosis induced by fructose. Such a scenario might explain why liver damage appears more quickly in fructose-fed LCN2 KO mice. In line with this hypothesis, fructose-treated WT as well as LCN2 KO mice showed more and bigger fat droplets in Oil Red O-stained slices of their livers compared to controls (Figures 3A,B). In this case, female mice developed more steatosis than males, although LCN2 KO males suffered more damage than WT males. The highest grade of steatosis was obtained in female LCN2 KO mice that had received fructose-enriched chow for 8 weeks. These data, as well as our Western blot results, indicate that the uptake of fructose by the solid food route provokes more steatosis than fructose administered through drinking water.

Male WT mice exposed to fructose showed no upregulation of liver enzymes, indicating that their livers might be less susceptible to fructose treatment. In contrast, female WT mice showed a heightened increase in liver enzymes after consuming fructose-enriched chow for 8 weeks, suggesting that females are more prone to develop hepatic damage during prolonged fructose treatment. Remarkably, all LCN2-deficient female mice had elevated liver enzymes in comparison to WT controls, even when fed standard chow. However, no significant differences between the genotypes were measurable due high standard deviations.

### Steatosis is not caused by *de novo* lipogenesis

Exposure of the liver to high amounts of fructose triggers the expression of genes responsible for *de novo* lipogenesis. During this process, lipids are built from dietary sugars such as fructose in three consecutive steps. Fatty acids are first synthesized, then elongated or desaturated, and finally esterified with glycerol and bundled to form TG (Softic et al., 2016). Unlike glucose, fructose uptake is not regulated by insulin. When uncontrolled, fructose is metabolized to pyruvate and provides substrates for lipogenesis such as acetyl-CoA.

Hepatic SREBP is an important transcription factor regulating fatty acid synthesis (Basciano et al., 2005; Softic et al., 2016). SREBP, which is induced by fructose, binds to the SRE in the promoters of lipogenic genes and activates those genes (Koshy et al., 2015). In our study, the expression of SREBP-1c was upregulated much more in mice that received fructose in their food rather than in their water (Tables 3, 5). Although SREBP-1c mRNA levels were higher in LCN2 KO control mice compared to WT controls, fructose exposure caused significant differences within both the LCN2 KO female and LCN2 KO male groups. This effect was not observed in WT mice, suggesting that fructose provokes more effects in the absence of LCN2.

An initial step in the building of fatty acids is the production of malonyl-CoA by ACCα and ACCβ. Although fructose treatment tended to increase ACC expression in both WT and KO animals, only LCN2 KO mice showed a significant upregulation of this enzyme following fructose exposure. This difference was not noticed in WT mice, supporting our previous observation of higher vulnerability of *Lcn2*-deficient mice. Standard deviations were also lower in the mutant mice. Surprisingly, there was no difference in the effect on ACC between the feeding of fructose via food vs. water, and no additional increase in ACC expression with prolonged fructose exposure, suggesting that the observed rise in ACC is not induced by SREBP-1c. In contrast, the genotype had a significant effect on ACC during fructose exposure.

In another step of *de novo* lipogenesis, FAS uses malonyl-CoA to build up the saturated fatty acid palmitate. Dietary carbohydrates, and especially fructose, are known to increase mRNA levels of FAS (Basciano et al., 2005). Based on our observation showing that ACC activity was higher in fructose-treated mice, it can be assumed that there might be enough substrate for FAS to generate fatty acids. Most interestingly, the expression of FAS mRNA showed a trend towards suppression in nearly all fructose-treated mice. Furthermore, FAS mRNA expression dropped during fructose treatment in WT and KO. This finding was more pronounced in female mice. Under the experimental setting, high fructose may contribute to a process that hinders the progress of *de novo* lipogenesis and fatty acid production that can be observed in both sexes and genotypes.

The final steps of TG production are complex requiring fatty acid desaturation and elongation in order to be esterified to glycerophosphates. The desaturation process is catalyzed by SCD1, which converts saturated fatty acids to mono-unsaturated fatty acids.

In our study, fructose treatment had no prominent effect on *Scd1* expression. WT females that received fructose for 4 weeks tended to have increased *Scd1* expression, while prolonged treatment resulted in a marked decrease, suggesting a potential time-dependent adaptation process. In general, LCN2 KO mice exhibited less *Scd1* mRNA than WT mice. DGAT enzymes catalyzing the final step of *de novo* lipogenesis that leads to TG biosynthesis contribute to steatosis. As there was no significant upregulation in DGAT mRNA we suggest no production of TG and therefore no *de novo* lipogenesis during our experiment.

We also examined lipolytic markers in order to determine if fructose might inhibit the lipolysis of fat. CPT1, an enzyme involved in fatty acid transport that also initiates fat utilization in the mitochondria showed no alterations. If FFA are not removed via transport to the mitochondria, they can be esterified to build up TG, which then are stored in the liver. This TG accumulation also leads to the suppression of lipolytic enzymes, as exemplified by HSL, which tended be suppressed in fructose-treated males (Supplemental Figure 2). HSL mRNA expression was significantly lower in LCN2 KO male mice compared to WT male mice, but only the WT males showed a significant downregulation of HSL mRNA during fructose treatment. Fructose-exposed female WT mice displayed a tendency to enhanced HSL expression but with high variability. This variability might be explained by our decision not to incorporate a fasting period before fructose treatment sacrifice. In contrast, LCN2 KO females did not show this variation in HSL expression, confirming that LCN2 is involved in lipid homeostasis and its absence disturbs fat metabolism.

LXRα is a nuclear receptor that is assumed to promote cholesterol and glucose metabolism and to support fatty acid and TG synthesis. As such, LXRα appears to have a fundamental role in liver homeostasis (Zhao et al., 2012). In our study, LXRα expression was not altered in any fructose-exposed group, suggesting that fructose treatment does not have a significant effect on liver homeostasis. Therefore, it is likely that fructose exposure does not affect the initial steps of *de novo* lipogenesis but abrogates the final steps of fatty acid production. This effect might be due to the fact that fructose is a sugar that constrains lipogenesis and fatty acid production in healthy subjects. Fructose may therefore have an influence on fatty acid uptake by the liver and may promote hepatic fat accumulation rather than utilization or storage in adipose tissue.

Recent studies have reported that a prolonged diet of high fructose combined with high fat induces steatohepatitis, fibrosis and severe liver damage in mice (Leung et al., 2016; Baena et al., 2017; Love et al., 2017). While mice administered only liquid fructose did not show hypertriglyceridemia, a combination of high fat and high fructose induced TG accumulation, diminished insulin signaling, and increased cholesterol deposition. Moreover, this combination affected the whole body and triggered insulin resistance (Baena et al., 2017). In our study, most genetic markers involved in fat metabolism (except DGAT enzymes and SCD1) showed a significant difference between WT and LCN2 KO mice during fructose treatment, in line with LCN2's involvement in fat metabolism. In mice that lack LCN2 and thus struggle to control their fat metabolism, fructose supplementation potentiates the development of NAFLD.

With regard to serum glucose, our female WT mice developed a continuous increase in this parameter upon fructose treatment that was absent in LCN2-deficient mice (Figure 5A). In this context, it should be noted that, unlike humans, mice have capacity to transform fructose to glucose in the gut (Mayes, 1993). If this process is diminished by LCN2 deficiency, fructose could accumulate and be taken up by the liver.

In our study, steatosis occurred in response to fructose treatment, especially in female and LCN2 deficient mice. However, expression of genes involved in different steps of *de novo* lipogenesis were not upregulated consequently during fructose treatment (Table 5). Therefore, we presumed that fructose does not induce *de novo* lipogenesis in our study. Similarly, recent studies confirmed this finding (Jatkar et al., 2017) and not even found an induction of TG deposition or augmentation of epididymal fat (Ozawa et al., 2016). Moreover, another report showed fructose to induce steatohepatitis and fibrosis only when combined with trans-fatty acids, while saturated fatty acids in combination with fructose only provoked simple steatosis (Jeyapal et al., 2017). In this context, it is noticeable that fructose can worsen the outcome of liver damage by affecting dysbiosis in microbiota, impacting the immune system, and progression of NAFLD (Lambertz et al., 2017).

### LCN2 and estrogen interact in fat metabolism

It is well known that estrogens regulate adipose tissue development and fat deposition and degradation in females. Gou et al. reported that the expression of estrogen-regulated genes involved in cholesterol homeostasis is diminished in LCN2-deficient mice (Guo et al., 2016). LCN2 has a tissue-specific role in the biosynthesis of estradiol, which links LCN2 to obesity and metabolic complications. Female LCN2-deficient mice showed a reduction in 17β-estradiol when challenged with a high fat diet leading to metabolic disruptions (Guo et al., 2016). In male mice, a lack of LCN2 has an impact on energy metabolism, causing increased adiposity, adipocyte hypertrophy, dyslipidemia, and fatty liver. These results support the notion that LCN2 and estrogens have overlapping physiological functions (Guo et al., 2016), a hypothesis supported by our finding showing increased LCN2 expression in fructose-treated female mice (Figure 6A). As well, a study by Drew and coworkers described an interaction between LCN2 and estrogen receptor α wherein deletion of this receptor influenced adipocyte function and LCN2 expression in female mice (Drew et al., 2015). In this study, LCN2 expression was highly elevated when receptor levels were diminished. Therefore, it was suggested that the estrogen receptor is a transcriptional regulator of LCN2 that promotes tumorigenesis in the context of obesity (Drew et al., 2015). Since LCN2 regulates hepatic lipid uptake, this finding also explains our observation that fructose-exposed LCN2 KO females suffer from more severe steatosis than do WT females (Figure 3A).

### Concluding remarks on LCN2 in NAFLD

We conclude that the feeding of a high fructose diet to mice leads to hepatic steatosis. In particular, mice lacking LCN2 are more prone to hepatic insult during extended periods of fructose feeding. Feeding of chow containing 60% fructose causes more severe fat droplet formation in the liver than does the consumption of drinking water containing 30% fructose. This result is somewhat surprising because the latter group of mice actually receives more energy from their diet and thus is able to potentially produce more fat.

Our work also shows that fructose-induced liver steatosis does not arise from *de novo* lipogenesis. Instead, fructose appears to directly affect liver homeostasis, thereby manipulating fat metabolism. Female mice showed higher steatosis and damage after fructose treatment than their male counterparts, confirming the potential influence of estrogen on lipid homeostasis reported in previous studies. Furthermore, compared to WT animals, LCN2 KO mice develop more hepatic steatosis after fructose treatment. We have previously reported that LCN2-deficient mice are more prone to the development of fatty livers even when fed standard chow. Therefore, we speculated that LCN2 is directly or indirectly involved in hepatic lipid uptake. The mice used in our study lacked LCN2 in all organs and tissues. Therefore, the observed effects might originate from different LCN2 attributes or are provoked by a sum of different organ-specific or -unspecific activities. Fructose might disturb liver homeostasis by promoting lipid uptake into the liver, while LCN2 counteracts this lipid uptake. One important limitation of our study is the fact that the mice were not fasted before sacrifice, resulting in values with high standard deviations that complicated assessments of differences between groups. Furthermore, treatment for 4 or 8 weeks might still be too short to unravel the complete pathogenesis of fructose-induced liver damage. A longer treatment period might provoke stable stimulation of lipogenesis and greater liver damage. Nevertheless, our results illustrate the tremendous deterious effects of consuming high amounts of fructose. Such effects should be of concern to humans consuming beverages containing high fructose corn syrup, a common sweetener. Further studies focusing on the mechanisms underlying fructose-induced pathogenesis of NAFLD, the biological functions of LCN2, and the modulation of these processes by estrogen could potentially lead to novel treatment strategies that will help to decrease the number of patients suffering from elevated hepatic fat deposition and other fat-related disorders.

## Author contributions

JL: performance of measurements, data compiling and writing of manuscript; TB: supply of *Lcn2* deficient mice and discussion of results; TM: supply of *Lcn2* deficient mice; JvH: measurement of serum parameters; RW: design of study, experimentation, and writing of manuscript.

### Conflict of interest statement

The authors declare that the research was conducted in the absence of any commercial or financial relationships that could be construed as a potential conflict of interest.

## Abbreviations

ACCα/β: Acetyl-CoA carboxylase α or β
ALT: alanine aminotransferase
apoB: apolipoprotein B-100
AST: aspartate-aminotransferase
ATGL: adipose triglyceride synthase
Co: control(s)
CPT1: carnitine palmitoyltransferase 1
DGAT1/2: diacylglycerol acyltransferase1 or 2
ER: endoplasmatic reticulum
FABP5: fatty acid binding protein 5
FAS: fatty acid synthase
FF: animal group fed a sugar-free drinking water and a special fructose-containing chow
FFA: free fatty acid(s)
FW: animal group fed a standard chow supplemented with 30% (w/v) fructose in drinking water
HSL: hormone-sensitive lipase
IR: insulin receptor
KO: knockout
LCN2: lipocalin 2
LXRα: liver X receptor α
NAFLD: non-alcoholic fatty liver disease
NASH: non-alcoholic steatohepatitis
NEFA: non-essential fatty acid(s)
PLIN5: perilipin 5
SCD1: stearoyl-CoA desaturase 1
SRE: sterol-responsive element(s)
SREBP-1c: sterol regulatory element-binding transcription factor 1c
TAG: triacylglycerol
TG: triglycerides
VLDL: very low density lipoprotein
WT: wild type.
