# Supplementary material for: Lipocalin-2 in Fructose-Induced Fatty Liver Disease
Source: Front Physiol. 2017 Nov 28;8:964. doi: 10.3389/fphys.2017.00964 (PMC5712346; doi:10.3389/fphys.2017.00964)
Supplement: Supplementary file 1 [file DataSheet1.DOC]

**Legends to the Supplemental Figures**

**Supplemental Figure 1: Feeding scheme.** WT and *Lcn2*-deficient mice were fed for 4 or 8 weeks with fructose-enriched diets administered either through drinking water (30%) or chow enriched with 60% fructose. Body weights and food intake was measured regularly. After sacrifice, the liver weights were determined, extracts for Western blot and qRT-PCR analysis prepared, and material for immunohistochemical analysis snap frozen.

**Supplemental Figure 2: Hepatic expression of genes involved in fatty acid synthesis and oxidation.** Relative mRNA expression of **(A)** SREBP-1c, **(B)** Accα **(C)** Accβ, **(D)** FAS, **(E)** SCD1, **(F)** DGAT1, **(G)** DGAT2, **(H)** CPT1, **(I)** HSL, and **(J)** LXRα were measured by qRT-PCR. All values were normalized to GAPDH content. Significant expression differences between groups found in ANOVA testing are * p > 0.05 and ** p > 0.01.
