# Supplementary figures and images for: Lipocalin-2 in Fructose-Induced Fatty Liver Disease
Source: Front Physiol. 2017 Nov 28;8:964. doi: 10.3389/fphys.2017.00964 (PMC5712346; doi:10.3389/fphys.2017.00964)

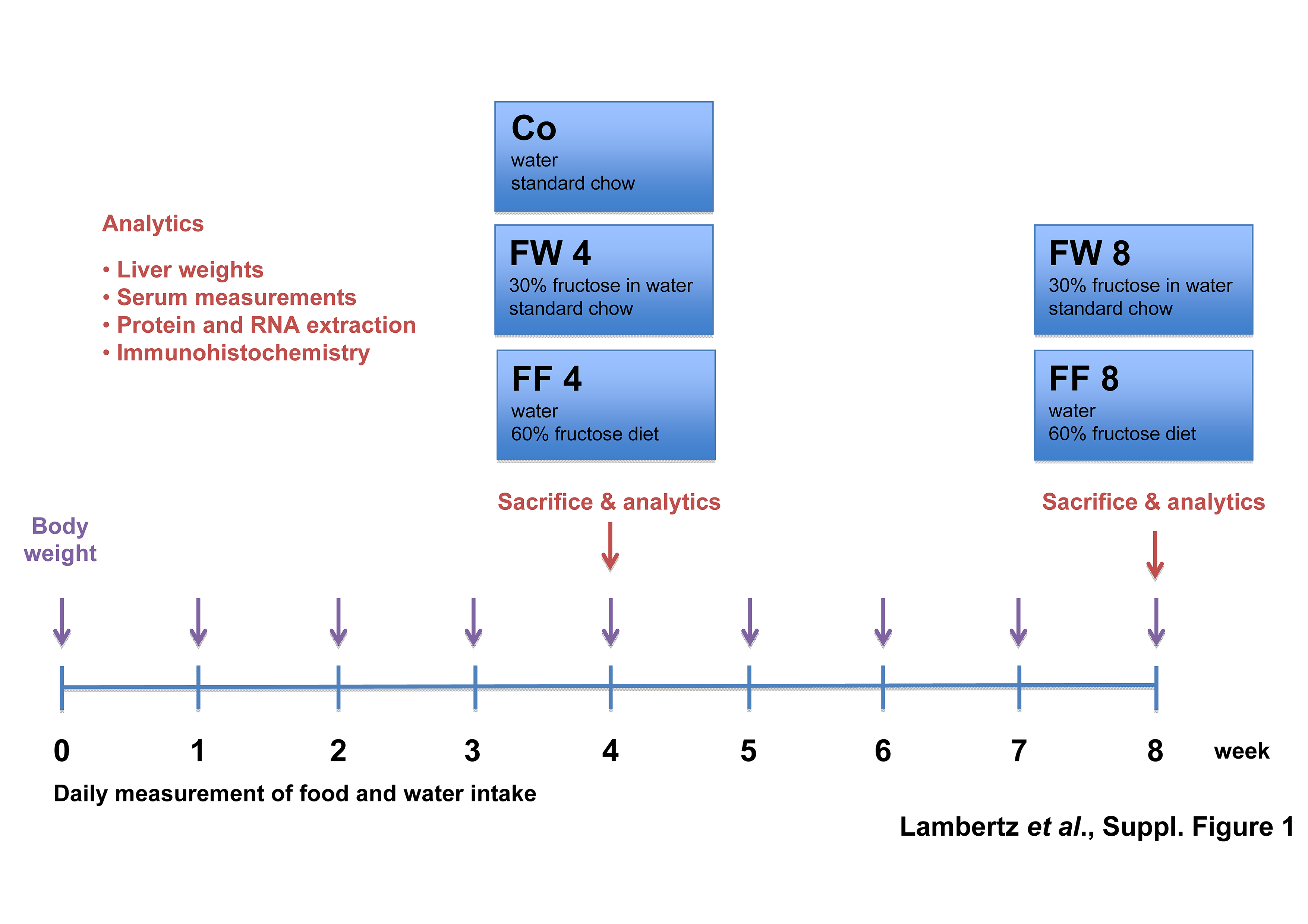

Supplement: Supplementary file 2 [file Image1.TIFF]

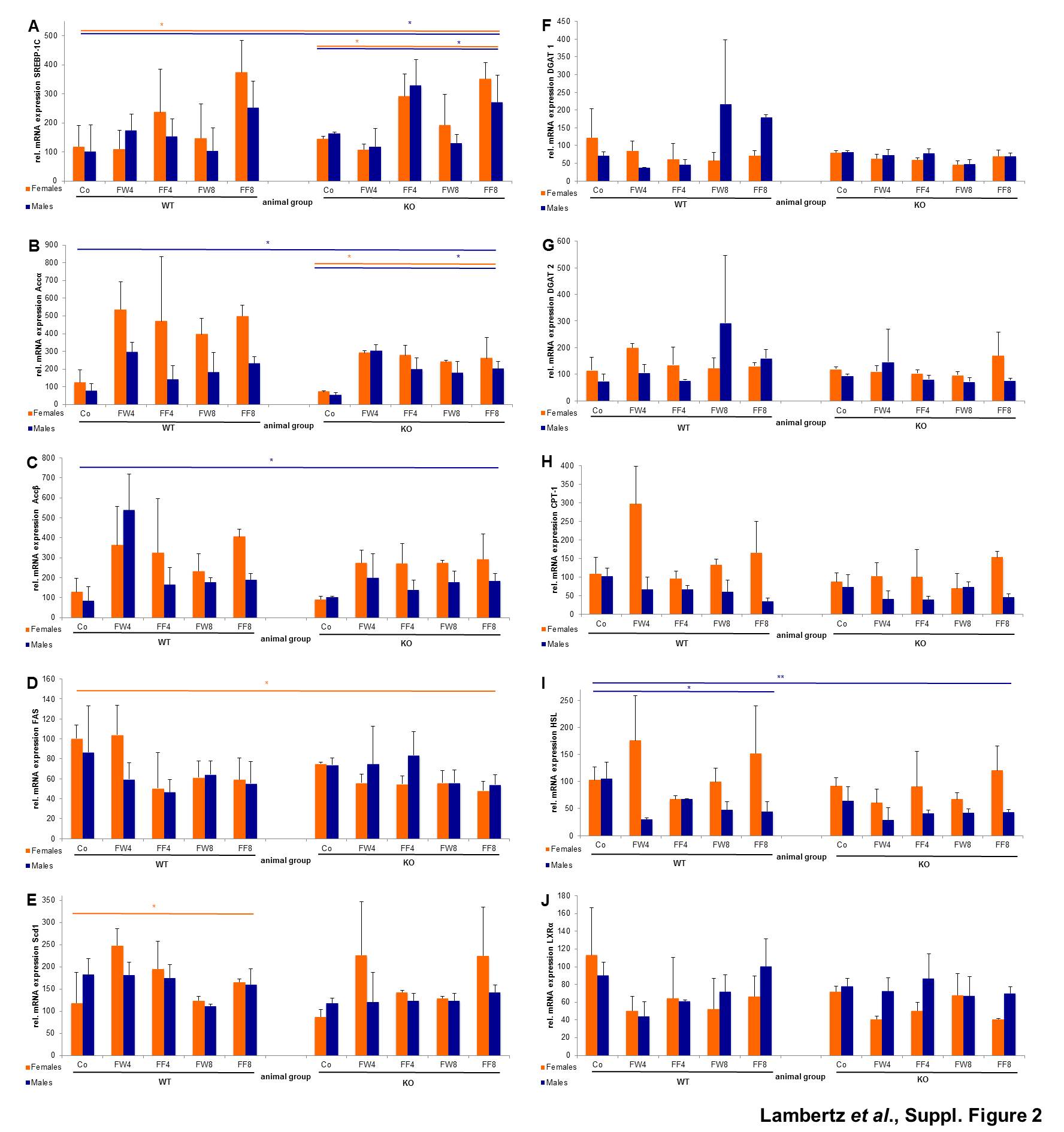

Supplement: Supplementary file 3 [file Image2.jpg]
